# Synthesis of Polyhydroxyurethanes—Experimental Verification of the Box–Behnken Optimization Model

**DOI:** 10.3390/polym14214510

**Published:** 2022-10-25

**Authors:** Michał J. Zalewski, Mariusz Ł. Mamiński, Paweł G. Parzuchowski

**Affiliations:** 1 Faculty of Chemistry, Warsaw University of Technology, 3 Noakowskiego St., 00-664 Warsaw, Poland; 2 Institute of Wood Sciences and Furniture, Warsaw University of Life Sciences–WULS, 159 Nowoursynowska St., 02-776 Warsaw, Poland

**Keywords:** PHU, polyhydroxyurethane, Box–Behnken design, model, optimization

## Abstract

Polyurethanes are one of the most important groups of polymers for numerous sectors of industry. Their production involves using dangerous components (diisocyanates), thus, in the search for safer synthetic routes, alternative methods yielding non-isocyanate polyurethanes (NIPU) have been investigated. In this study, the synthesis of polyhydroxyurethane from cyclic carbonates was performed. A three-factor, three-level Box–Behnken experimental design was constructed and the reaction time, temperature and reagents’ molar ratio were the independent variables. The built model revealed that the viscosity was influenced by all three independent factors, while the mechanical properties and glass transition temperature of the PHUs were affected by the reagents’ ratios. An experimental verification of the model proved its accuracy as the mechanical strength and glass transition temperature deviated from the modeled values, by 15% and 7%, respectively.

## 1. Introduction

Polyurethanes are one of the most important groups of polymers in terms of global production and number of possible applications. The conventional synthesis of PUs uses a step growth polyaddition reaction of polyols with diisocyanates. One of these components, the isocyanates (mainly toluene diisocyanate—TDI and methanediphenyl diisocyanate—MDI) are harmful, carcinogenic and have mutagenic compounds. Moreover, their production involves extremely toxic gas—phosgene, which makes the process hazardous to the environment and to human health [1]. Regardless of those drawbacks, polyurethane materials are widely used in many industrial applications such as the automotive industry, the construction industry, thermal and sound insulation, thermoplastics, textiles, adhesives and coatings [2]. On the other hand, keeping in mind the principles of green chemistry that recommend substitution of harmful raw materials for non-harmful ones wherever technically possible [3], alternative methods of non-isocyanate polyurethanes (NIPU) synthesis via isocyanate-free routes have been described in the literature. Synthesis of NIPU can employ: (1) “AB-type” precursors bearing both hydroxyl and reactive functional groups in the same molecule [4], (2) reaction of alkylene bis(chloroformate)s with diamines [5]; (3) reaction of alkylene bis(phenyl carbonate) with diamine [6].

Additionally, more recent approaches to NIPU are based on (1) thiol-ene self-photopolymerization [7], (2) synthesis from α, ω-aminoalcohols [8], (3) self-polycondensation reaction of hydroxyl-acyl azide moiety [9], or (4) polycondensation of respective dicarbamates with diols [10]. A consecutive search for the greener ways to NIPU provided the polycondensation of bis(methyl dicarbamate)s with diols [11,12] and a specially useful and environmentally benign approach—copolymerization of cyclic carbonates with amine-bearing compounds [13].

What is interesting is that reactions of amines and bis(cyclic carbonate)s can also lead to polyhydroxyurethanes (PHU)—a new class of NIPU that has been paid much attention in recent years due to its environmental benignancy and excellent mechanical properties resulting from the presence of intra- and inter-molecular hydrogen bonding of hydroxyl and carbonyl groups in the polymer structure [14].

Although the industrial application of PHU greatly covers those of the traditional polyurethanes, their synthesis strategy is more convenient, safer and atom-economic. These are (1) the polyaddition of poly(cyclic carbonate)s with polyamines [15], and (2) polyaddition of glycerol-derived carbonates with amines [16,17,18], to name just a few.

A comprehensive review of the synthesis and application of non-isocyanate polyurethanes (NIPU) can be found elsewhere [19].

Cramail et al. analyzed the effect of substitution degree in amine and cyclic carbonates and the temperature on the reaction rate. They found that the degree of substitution of cyclic carbonate lowered the reaction rate towards the amine as well as revealed that internal cyclic carbonate reaction rates increased when the temperature increased [20], however, excessive temperature increase favored substituted urea formation resulting from the subsequent attack of another amine molecule [21]. Webster and Crain found that initial the concentration of amine significantly affected the reaction rate [22]. 

Hence, the efficient polyaddition and molar mass of the product are determined by the stoichiometric ratio of reagents, the temperature and the time of reaction. The Box–Behnken design is often used in the chemistry of polyurethanes [23,24,25,26]. 

The aim of this study was the optimization of polyhydroxyurethane synthesis under various conditions according to the three-factor, three-level Box–Behnken experimental plan. The design comprised 15 experimental runs that reflect points positioned at the midpoint of edges of the multi-dimensional cube and the replicated center point in it [27,28].

The variables were reaction time, temperature and the molar ratio of bis(cyclic carbonate)s. Based on the physiochemical properties of the obtained polymers, a mathematical model was made. Then the model was empirically verified.

## 2. Materials and Methods

The 1,6-hexamethylenediamine (HMDA) (99%) was purchased from Sigma-Aldrich (Poznań, Poland) and used as received. Syntheses of bis(glycerol carbonate) (BGC) and bisphenol A dicarbonate (DCBPA) were performed according to the previously described procedures [29,30].

### 2.1. PHUs Synthesis

Reagents in amounts as indicated in Table 1 and Table 2 were placed in a 250-mL three-necked flask equipped with a mechanical stirrer, a reflux condenser, a thermometer and an argon inlet. Reactions were performed under argon atmosphere. The temperature and time are shown in Table 3. The obtained viscous liquid was next transferred to a PE vial and cooled down to solidify at ambient temperature.

### 2.2. Instrumentation

FTIR (Fourier transform infrared) spectra were recorded on a Nicolet iS5 Mid Infrared FT-IR Spectrometer equipped with iD7 ATR optical base (Thermo Scientific^®^, Waltham, MA, USA). 1H NMR spectra were recorded on a Varian VXR 400 MHz (Palo Alto, Santa Clara, CA, USA) using tetramethyl silane as an internal standard. Samples of 85 mg were dissolved in deuterated DMSO-d6. DSC (Differential scanning calorimetry) measurements were carried out using a Q200 DSC (TA Instruments, New Castle, DE, USA). The heating rate was 10 °C min^−1^. The measurements were performed on 20 mg samples in closed aluminum trays in the constant flow of argon 10 mL min^−1^. Viscosity was measured on a Kinexus rheometer (Netzsch, Selb, Germany) with (8-mm diameter) plate–plate geometry at 140 °C and 1/min speed. Mechanical performance of the PHUs was measured on an INSTRON 5566 (Norwood, MA, USA).

### 2.3. Design of Experiments

Three independent factors were coded on three levels: −1 (low level), 0 (standard level, arithmetic mean of −1 and +1), and +1 (high level). The Box–Behnken design avoids experimental runs with all three factors set at their extremes. Such an approach allows for the determination of polynomial quadratic equations. A three-level, three-factor Box–Behnken design was established by the Design Expert^®^ software (version 6.0.10. Stat-Ease, Minneapolis, MN, USA). 

The 15 experiments constructed and performed are shown in Table 1. The three independent variables applied to investigate the responses were: reaction time, temperature and the molar ratio of two bis(cyclic carbonate)s, while viscosity (η), glass transition temperature (*T_g_*) and shear strength (*R_t_*) were the responses. The responses obtained from 15 experimental runs were fed back into Design Expert software to compute the polynomial equations for the interactions. Table 1, Table 2 and Table 3, present coded levels, actual levels for the applied factors, and experimental design matrix, respectively.

### 2.4. Mechanical Performance of PHUs

**Figure 1:**
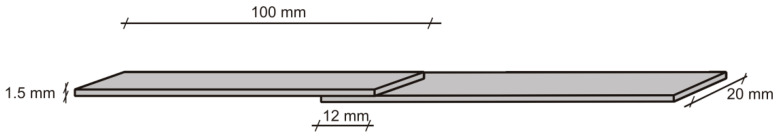
Lap specimen used in bondline strength measurements.

Mechanical strength of the obtained PHUs was tested as lap shear strength of the bondline in pinewood specimens of dimensions 100 mm × 20 mm ×1.5 mm (Figure 1). A 0.5-mm thick foil of a PHU was applied between two 1.5-mm thick veneers, so that a lap of the dimensions 20 mm × 12 mm was formed. Next, specimens were bonded in a hot press at 150 °C for 30 s under 0.8 MPa pressure, then immediately transferred to a cold press and kept for 5 min under 0.8 MPa pressure to cool down and set the bondline. Bonded specimens were conditioned at normal conditions (20 ± 2 °C and 65 ± 5% relative humidity) for 24 h before testing. Ten specimens were tested in each series. Shear speed was 20 mm/min.

Shear strength (*R_t_*) was calculated from the Equation (1):

(1) $$R_{t}=\frac{F_{max}}{S}$$

where: *F_max_* is the maximum force in Newtons and *S* is lap area in mm^2^.

## 3. Results

### 3.1. Box–Behnken Design

Box–Behnken design is a rotatable plan which means that the prediction variance depends only on the distance of the design point from the center of the design [31]. The greater the absolute value of the coded variable, the greater the variance of the calculated value. The variance is calculated from the last three experiments nos. 13, 14, and 15 in which the independent variables are kept constant at level 0, so the differences in the results of these experiments are not variable. Hence, the difference in the results of these experiments allows the variance to be calculated.

The low level for the reaction time was 60 min, because our initial experiments revealed that such time was sufficient for cyclic carbonates to converse completely, i.e., ~1800 cm^−1^ band disappeared on the FTIR spectrum. The high level was 120 min as a longer reaction time may cause allophanates formation. The standard level (0) was 90 min which is an arithmetic mean of high- and low-level values (Equation (2)).

(2) $$x=\frac{120+60}{2}=90\;\min$$

The low level for the reaction temperature was 120 °C due to the melting point of DCBPA [30]. The high level was 160 °C to avoid favorable allophanate formation at higher temperatures which might reduce the properties of a PHU. The standard level (0) was an arithmetic mean of high and low levels (Equation (3)).

(3) $$x=\frac{160+120}{2}=140\;{}^{\circ}C$$

All experimental runs were performed at constant 1,6-hexamethylenediamine content 0.10 moles, while the variable was BGC/DCBPA carbonates molar ratio. The high level (+1) was coded for 0.10 moles BGC, low level (−1) for 0.10 moles DCBPA and the standard level (0) was coded for 0.05 moles BGC: 0.05 moles DCBPA mixture.

### 3.2. PHUs Characterization

**Figure 2:**
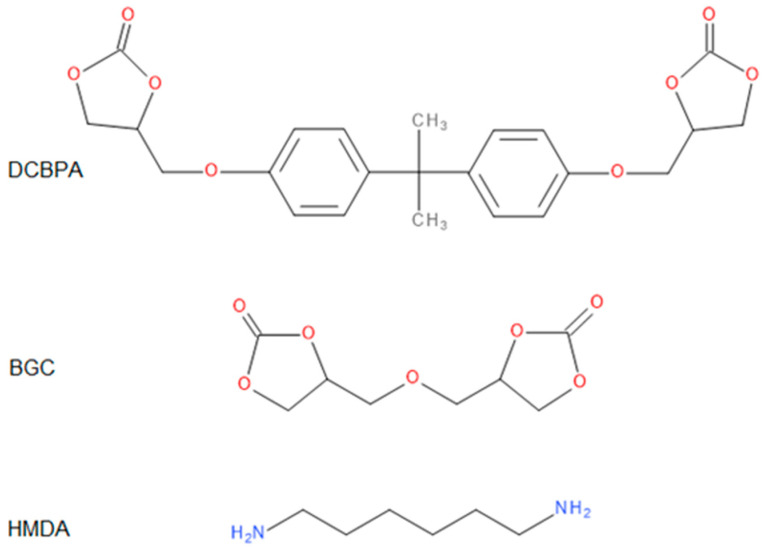
Structures of the reagents.

**Figure 3:**
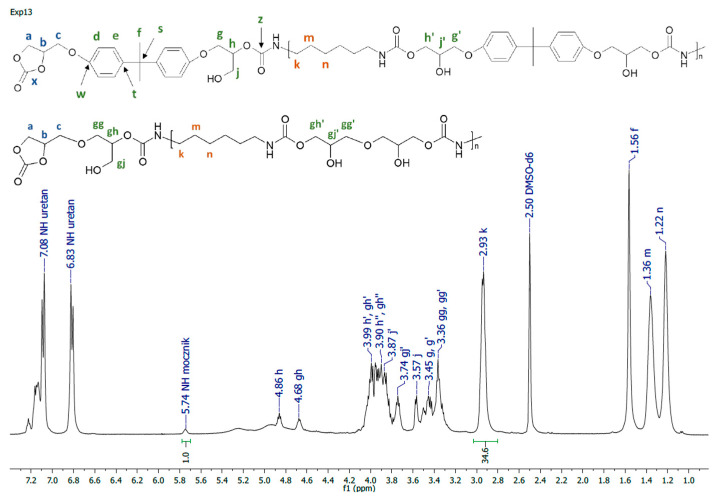
1H NMR spectrum of PHU synthesized in experiment run nos. 1–4, 13–15.

**Figure 4:**
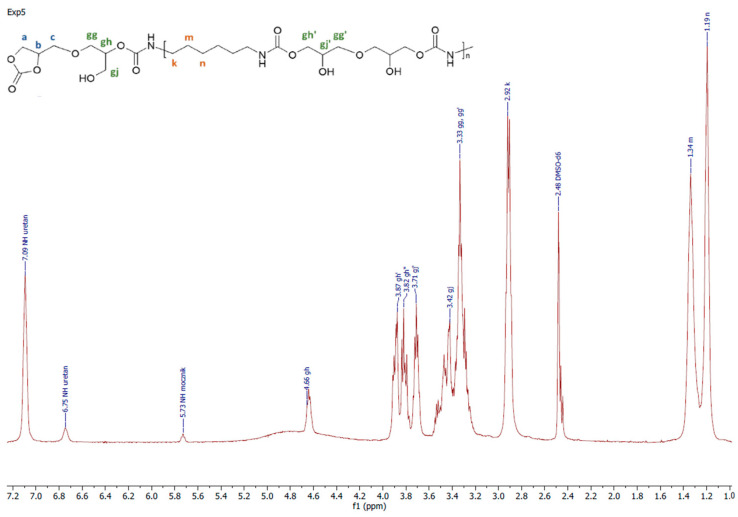
A representative 1H NMR spectrum of PHU obtained in experiment run nos. 5, 6, 9 and 10.

**Figure 5:**
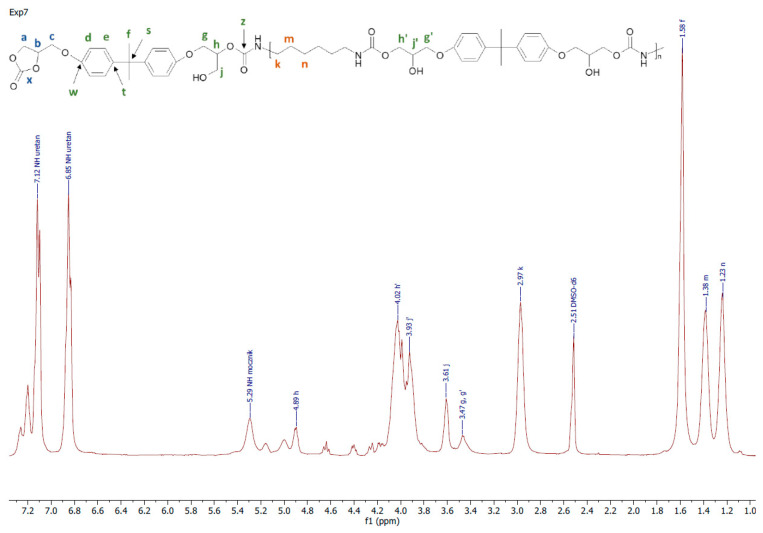
A representative 1H NMR spectrum of PHU obtained in experiment run nos. 7, 8, 11 and 12.

All the obtained polymers were solids. It is apparent that the carbonates molar ratio (factor 3) affected the PHUs macromolecule structures, and subsequently, their physicochemical properties were different. The reagents’ structures are shown in Figure 2. Actual structures of the obtained PHUs were elucidated by 1H NMR in Figure 3, Figure 4 and Figure 5.

A representative 1H NMR spectrum and macromolecular structure of the PHUs obtained in the runs 1–4 and 13–15, where carbonate mixture composition was kept constant, are presented in Figure 3. The polyurethanes synthesized in the run nos. 5, 6, 9 and 10 at a constant amount of 0.10 moles BGC. 1H NMR spectrum and the molecular structure were presented in Figure 4. In Figure 5, the structure and 1H NMR spectrum of PHU from experiment run nos. 7, 8, 11 and 12 (0.10 moles DCBPA) were shown. All the synthesized PHUs were transparent-to-opaque yellow-green solids. 

### 3.3. Mechanical Performance

**Figure 6:**
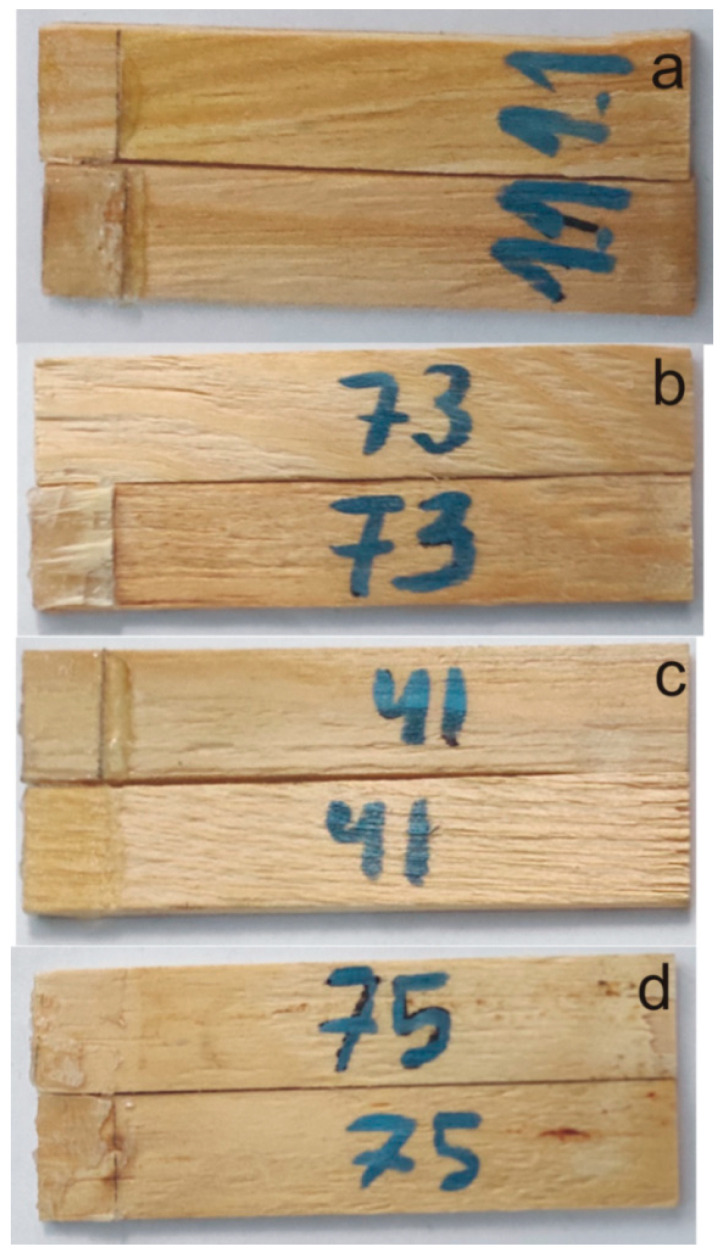
Failure modes observed in the tested specimens: (**a**)—adhesive at the binder/wood interface, (**b**)—cohesive in wood, (**c**)—cohesive in PHU layer, and (**d**)—mixed.

The mechanical performance of the PHUs was investigated on the basis of the bonding strength of bondlines in solid wood. Lap shear strengths are presented in Table 4. The variations in the observed strengths result from variations in failure modes: (i) adhesive, (ii) cohesive in adhesive layer, (iii) cohesive in wood or (iv) mixed [2]. Respective failure modes appeared in all the series, regardless of the PHU synthesis conditions (Figure 6). Thus, the determined strengths are averaged, but the values are still in the range typical for thermoplastic adhesives: poly(vinyl acetate) 0.5–3.7 MPa [32], poly(oxetane)s 0.4–1.3 MPa [33] or poly(lactide)-poly(caprolactone)-based and EVA (0.6–1.5 MPa) [34] or HDPE 1.6–2.9 MPa [35]. The computed variation coefficients for the respective series remain at the levels comparable to those found in the literature for hot-melt adhesives (i.e., 20–30%) [36].

### 3.4. Thermal Properties

**Figure 7:**
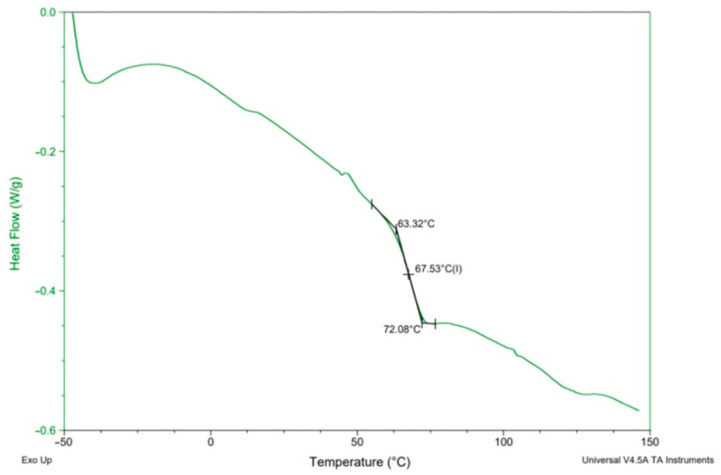
An exemplary DSC curve of PHU run no. 11.

**Figure 8:**
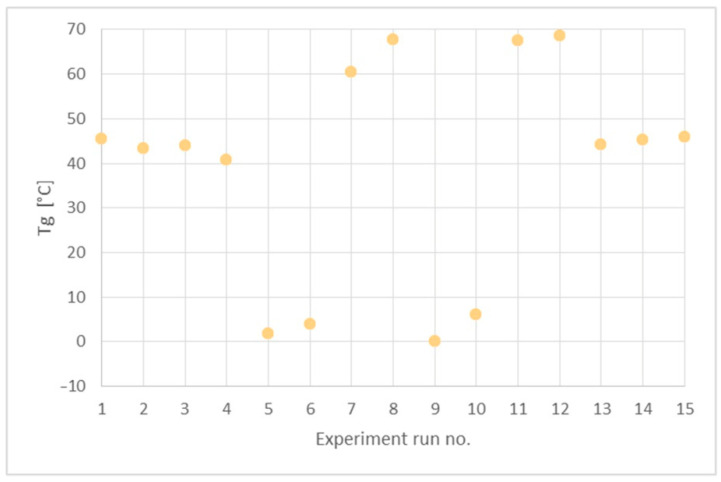
Glass transition temperatures (*T_g_*) of the obtained PHUs.

In order to determine the synthesis conditions on the thermal properties of the PHUs, the polymers were subjected to DSC analysis. Measurements were performed in the range −50 to 150 °C. Glass transition temperatures (*T_g_*s) are presented in Table 5. A DSC curve recorded for the PHU synthesized in experiment no. 11 is shown in Figure 7, while *T_g_*s of all the obtained PHUs are combined in Figure 8. In the applied temperature range only glass transition occurred in all cases, so it proves that the PHUs are amorphous.

The data in Table 5 and Figure 8 indicate three types of the polymers resulting from the reagents’ compositions. The first group are thermoplastics synthesized in run nos. 5 (120 °C, 90 min), 6 (160 °C, 90 min), 9 (140 °C, 60 min), 10 (140 °C, 120 min), where DCBPA was used, which exhibit *T_g_*s in the range between 0 °C and 10 °C. What is worth noting is that both the temperature increases and prolonged reaction time resulted in just slight increase in the *T_g_*.

The experiment run nos. 1 (120 °C, 60 min), 2 (160 °C, 60 min), 3 (120 °C, 120 min), 4 (160 °C, 120 min), 13, 14 and 15 (140 °C, 90 min) with equimolar mixture of BGC/DCBPA yielded PHUs of *T_g_*s between 40 °C and 50 °C. The effect of reaction temperature increase resulted in a slight decrease in the *T_g_* of the product.

The highest *T_g_*s (60–70 °C) exhibited the polymers obtained in the run nos. 7 (120 °C, 90 min), 8 (160 °C, 90 min), 11 (140 °C, 60 min) and 12 (140 °C, 120 min). Apparently, the significant factor affecting *T_g_* was the temperature, while reaction time was insignificant. 

As the lowest *T_g_*s were obtained for purely aliphatic PHUs, the above results are coherent with the commonly agreed knowledge on the influence of polymer structure on its thermal properties.

### 3.5. Viscosity

Due to the presence of hydroxyl groups in macromolecules, PHUs easily absorb moisture from the air, thus, prior to rheological measurements samples were dried at 50 °C in vacuum. Data collected in Table 5 shows that PHUs from run nos. 7, 8, 11 and 12 exhibited outstandingly high viscosities. A common trait for those polymers is the composition, i.e., the presence of aromatic DCBPA, which apparently contributes to inter- and intra-molecular interactions (i.e., π–π stacking), and brings hard and rigid segments, that subsequently increase the viscosity [37,38]. The observation is also in agreement with studies on the influence of aromatic moieties on the polymer viscosity. Mamiński et al. showed that the viscosity of a bisphenol A-cored polyglycerol with 2, 5 and 10 glycerol carbonate residues were, respectively, 430 Pa·s, 55 Pa·s and 18 Pa·s at 23 °C, which demonstrates that aromatic–aromatic interactions weaken when hindered [39]. On the other hand, the PHUs of purely aliphatic backbone or synthesized from equimolar mixture of BGC/CER exhibited 10–50 times lower viscosity. The phenomenon can be explained by either a plasticizing effect of BGC or remote positions of aromatic rings. On the other hand, the PHUs of purely aliphatic backbone or synthesized from an equimolar mixture of BGC/DCBPA exhibited 10–50 times lower viscosity. The phenomenon can be explained by either a plasticizing effect of BGC and/or remote positions of aromatic rings in the molecule.

### 3.6. Mathematical Relations between Variables

This work is focused on the analysis of how reaction time, temperature and reagents’ molar ratios affect the properties of products, i.e., PHUs. The Box–Behnken design provides a mathematical relation between the variables in the form of polynomial a quadratic equation (Equation (4)).

The polynomial equation for all the individual responses in terms of coded independent factors is as follows:

(4) $$y=b_{0}+\sum_{i=1}^{k}b_{i}x_{i}+\sum_{i=1}^{k}\sum_{j=1}^{k}b_{ij}x_{i}x_{j}+e_{i}$$

where: y is the computed response of the system, b is the coefficient of the equation, *b*_0_ is the free term, x is the independent variable, k is the number of the independent variables, and *e* is the constant.

The free term *b*_0_ is computed from Equation (5) as the arithmetic mean of values of a given trait, while Equation (6) describes determination of the coefficients in monomials bearing one independent variable in power of one. Equation (7) is used in determination of the coefficients in monomials bearing one independent variable in power of two, and Equation (8) concerns determination of the coefficients in monomials with the product of two independent variables in power of one. The approach yields the interactions between variables.

(5) $$b_{0}={\bar{y}}_{0}$$

(6) $$b_{i}=A\sum_{u=1}^{N}x_{iu}y_{ui}$$

(7) $$b_{ii}=B\sum_{u=1}^{N}x_{iu}^{2}y_{u}+C_{1}\sum_{j=1}^{k}\sum_{u=1}^{N}x_{ju}^{2}y_{u}-\frac{{\bar{y}}_{0}}{w}$$

(8) $$b_{ij}=D_{1}\sum_{u=1}^{N}x_{iu}x_{ju}y_{u}$$

where ${\bar{y}}_{0}$ is the arithmetic mean of *y* results of the population when coded factors are equal 0; N is the experiment number (population); $A,B,C_{1},D_{1},w$ is the constants as shown in Table 6.

The Box–Behnken design allows computing the variations for the determined correlation coefficients. Equation (9) yields the variation for the free term in Equation (5), Equation (10) yields coefficients of the monomials in Equation (6), while Equations (11) and (12) yield the coefficients determined in Equations (7) and (8), respectively.

(9) $$s_{b_{0}}^{2}=\frac{s_{rep}^{2}}{n_{0}}$$

(10) $$s_{b_{i}}^{2}=As_{rep}^{2}$$

(11) $$s_{b_{ii}}^{2}=\left(B+\frac{1}{w^{2}n_{0}}\right)s_{rep}^{2}$$

(12) $$s_{b_{ij}}^{2}=D_{1}s_{rep}^{2}$$

where: $s_{rep}^{2}$ is the variation determined for *y* results of the population when the coded factors are equal 0, $n_{0}$ is the number of experiments when the coded factors are equal 0.

Next, the computed correlation coefficients and their variations were the subject of the null hypothesis (*H*_0_) test (Equation (13)) against the alternative hypothesis (*H_a_*) (Equation (14)). For each of the coefficients $t_{calc}$ was computed (Equation (15)) and compared to $t_{crit}$ (Equation (16)). If $t_{crit}<t_{calc}$, then the null hypothesis was rejected, while *b*_i_ ≠ 0 condition was accepted at a 95% confidence level. Hence, the variable bound to this coefficient affects a given trait. However, if $t_{crit}>t_{calc},$ then the null hypothesis must not be rejected and *b*_i_ = 0. Then, it can be concluded at a 95% confidence level that the variable bound to this coefficient has no influence on a given trait.

(13) $$H_{0}:b_{i}=0$$

(14) $$H_{a}:b_{i}\neq 0$$

(15) $$t_{calc}=\frac{\left|b_{i}\right|}{s_{b_{i}}}$$

(16) $$t_{crit}\left(0.05;2\right)$$

The abovementioned approach was applied to determine the effect of the independent variables (reaction time, temperature and reagents’ molar ratio) on the properties of PHUs such as viscosity, glass transition temperature and mechanical performance.

**Figure 9:**
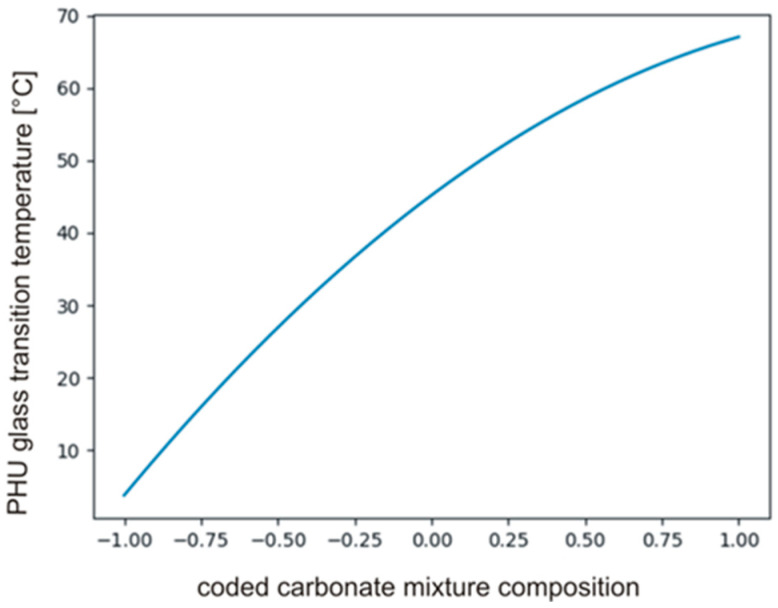
The plot of the function described by Equation (17).

The *t_crit_* was calculated using MS Excel T.INV function bounding probability (0.05) and degree of freedom (2), then $t_{crit}\left(0.05;2\right)=4.303$. The coefficients in polynomial describing *T_g_*’s and their significance are presented in Table 7. The data indicate that *b*_0_, *b*_3_ and *b*_33_ only are significant, hence, the carbonate used in synthesis had a significant effect on the *T_g_* of PHUs. The effect of the other variables, i.e., temperature (*b*_1_, *b*_11_) and time (*b*_2_, *b*_22_), was not significant. The null hypothesis was also tested for the monomials describing interactions of the variables (*b*_12_, *b*_13_, *b*_23_). Equation (17) describes relations between significant monomials, and Figure 9 shows the plot of the function in the range between −1 and +1.

(17) $$y=b_{0}+b_{3}x_{3}+b_{33}x_{3}=45.17+31.62x_{3}-9.78x_{3}^{2}$$

**Figure 10:**
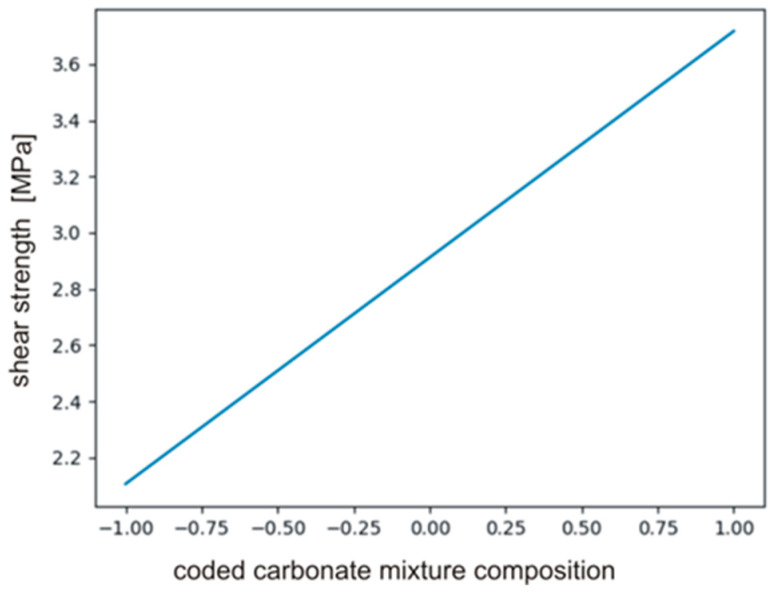
The plot of the function described by Equation (18).

The calculated coefficients of the polynomial describing the effect on PHUs mechanical properties are presented in Table 8. It is clear that only *b*_0_ and *b*_3_ had significant effect, i.e., composition of carbonate reagents (*b*_3_). The variables temperature (*b*_1_, *b*_11_) and time (*b*_2_, *b*_22_) were found to be insignificant. The null hypothesis was tested for the monomials describing interactions between the variables (*b*_12_, *b*_13_, *b*_23_). Equation (18) defines relations between significant monomials, and Figure 10 shows the plot of the function in the range between −1 and +1.

(18) $$y=b_{0}+b_{3}x_{3}=2.91+0.81x_{3}$$

**Figure 11:**
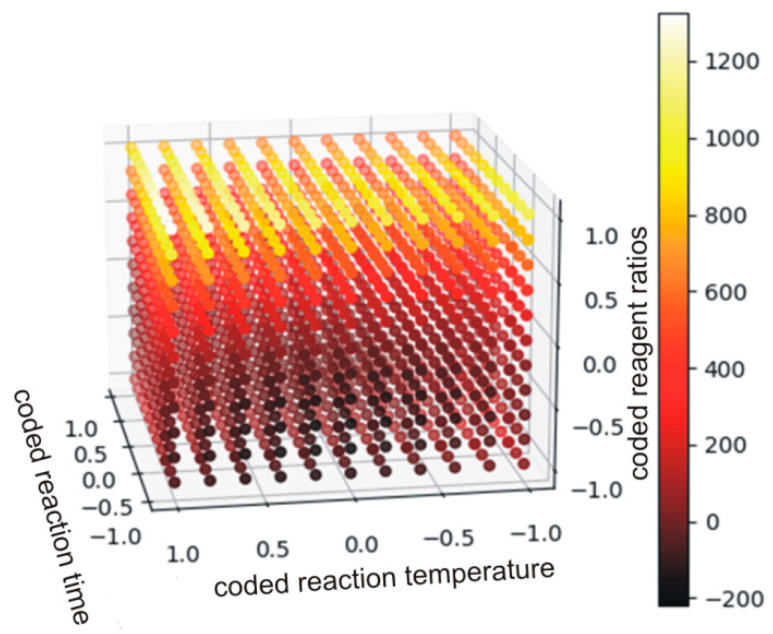
The plot of the function described by Equation (19) (the values below 0 have no physical meaning).

As the data in Table 9 indicate, the only insignificant monomial was found for the interaction of time and temperature (*b*_12_). For the other monomials the alternative hypothesis (*H_a_*) was accepted. The strongest effect on the viscosity was observed for the carbonate(s) used in the syntheses as the respective coefficients are significantly higher. Equation (19 defines relations between significant monomials, and Figure 11 shows the plot of the function in the range between −1 and +1.

Each point was described by 4 variables. The independent variables were ascribed to X, Y and Z axes, while viscosity was illustrated by the color. It is clear that the aromatic DCBPA (coded variable +1) yielded the PHUs of viscosities nearly two orders of magnitude higher when compared to other experiment runs. Additionally, coded variables at levels 0 and −1 result in viscosities differencing by decimals of order of magnitude. In such cases, a quadratic polynomial has an insufficiently flexible function to accurately model the existing relations.

(19) $$\begin{array}{c} y=b_{0}+b_{1}x_{1}+b_{2}x_{2}+b_{3}x_{3}+b_{13}x_{1}x_{3}+b_{23}x_{2}x_{3}+b_{11}x_{1}^{2}+b_{22}x_{2}^{2}+b_{33}x_{3}^{2}=45.52-78.67x_{1}\\ +43.32x_{2}+457.8x_{3}-172.98x_{1}x_{3}+96.17x_{2}x_{3}-114.82x_{1}^{2}+114.2x_{2}^{2}+433.29x_{3}^{2} \end{array}$$

In order to verify the accuracy of the model, synthesis under the optimized conditions was performed. Namely, temperature 120 °C, reaction time 78 min, and carbonate mixture composition: 0.06 moles DCBPA, 0.40 moles BGC and 0.10 moles HMDA. The true properties of the resultant PHU are compared with the modeled ones in Table 10.

As far as the shear strength and the *T_g_* are concerned, the values predicted by the model provided satisfactory coherence with the experiment. Lesser accordance was found for the viscosity. The gap might have been caused by a limited applicability of a quadratic polynomial to that specific interaction. One of the possible explanations is that the viscosity can be affected by moisture absorption [40].

## 4. Conclusions

A Box–Behnken optimization model was developed for the synthesis of polyhydroxyurethanes from 1,6-hexamethylenediamine, bis(glycerol carbonate) and bisphenol A dicarbonate. The mathematical model indicated that the viscosity was influenced by all three independent factors (reaction time, temperature and reagents’ molar ratio), while the mechanical properties and glass transition temperature of the PHUs were affected by reagents’ ratios only. This is coherent with the commonly recognized fact that the glass transition temperature is closely associated with the structure of the molecular chain.

The experimental verification of the accuracy of the developed model proved its satisfactory accuracy. Shear strength and glass transition temperature deviated from the computed values, by 15% and 7%, respectively, while the viscosity was the outlier.

## Figures and Tables

**Table 1:** Three-level factorial design in the Box–Behnken experimental plan.

Run No.	Factor 1	Factor 2	Factor 3
1	−1	−1	0
2	+1	−1	0
3	−1	+1	0
4	+1	+1	0
5	−1	0	−1
6	+1	0	−1
7	−1	0	+1
8	+1	0	+1
9	0	−1	−1
10	0	+1	−1
11	0	−1	+1
12	0	+1	+1
13	0	0	0
14	0	0	0
15	0	0	0

**Table 2:** Applied levels of the factors.

Coded Factor Level	Time [mins]	Temperature [°C]	Carbonate Molar Ratio
−1	60	120	0.10 BGC
0	90	140	0.05 BGC: 0.05 DCBPA
+1	120	160	0.10 DCBPA

**Table 3:** Experimental design matrix with actual levels of the factors applied in the experiments.

Run No.	Time [mins]	Temperature [°C]	Carbonate Molar Ratio
1	60	120	0.05 BGC: 0.05 DCBPA
2	60	160	0.05 BGC: 0.05 DCBPA
3	120	120	0.05 BGC: 0.05 DCBPA
4	120	160	0.05 BGC: 0.05 DCBPA
5	90	120	0.10 BGC
6	90	160	0.10 BGC
7	90	120	0.10 DCBPA
8	90	160	0.10 DCBPA
9	60	140	0.10 BGC
10	120	140	0.10 BGC
11	60	140	0.10 DCBPA
12	120	140	0.10 DCBPA
13	90	140	0.05 BGC: 0.05 DCBPA
14	90	140	0.05 BGC: 0.05 DCBPA
15	90	140	0.05 BGC: 0.05 DCBPA

**Table 4:** Lap shear strengths of PHUs bondlines in pinewood.

PHU No.	Shear Strength [MPa]	Standard Deviation [MPa]	Variation Coefficient [%]
1	2.31	0.38	16.4
2	2.27	0.38	16.7
3	2.50	0.69	27.6
4	2.72	0.72	26.5
5	1.43	0.32	22.4
6	1.69	0.43	25.4
7	3.32	0.87	26.2
8	2.67	0.78	29.2
9	1.01	0.61	0.60
10	1.91	0.64	33.5
11	2.13	0.86	40.4
12	3.10	0.30	9.7
13	3.32	0.85	25.6
14	3.44	0.70	20.3
15	3.19	0.64	20.0

**Table 5:** Glass transition temperatures (*T_g_*s) and viscosity at 140 °C for the investigated PHUs.

PHU No.	*T_g_* [°C]	Viscosity [mPa·s]
1	45.5	57
2	43.3	46
3	44.0	51
4	40.7	54
5	1.7	4
6	3.8	26
7	60.5	1020
8	67.8	378
9	0.0	15
10	6.0	9
11	67.5	984
12	68.6	1363
13	44.2	53
14	45.3	43
15	46.0	41

**Table 6:** The values of the constants.

Number of Variables	*A*	*B*	*C*_1_	*D*_1_	*w*
3	0.125	0.25	−0.0625	0.25	2

**Table 7:** The coefficients of polynomials found for *T_g_* and significance.

*b*_i_		*s_b_*	*t_calc_*	Significance
*b*_0_	45.17	0.50	90.16	+
*b*_1_	0.49	0.31	1.59	−
*b*_2_	0.37	0.31	1.20	−
*b*_3_	31.62	0.31	103.06	+
*b*_11_	−1.92	0.45	4.24	−
*b*_22_	0.12	0.45	0.26	−
*b*_33_	−9.78	0.45	21.65	+
*b*_12_	−0.27	0.43	0.61	−
*b*_13_	1.31	0.43	3.01	−
*b*_23_	−1.24	0.43	2.86	−

+ significant; − insignificant.

**Table 8:** The coefficients of polynomials found for mechanical properties of the PHUs.

*b*_i_		*s_b_*	*t_calc_*	Significance
*b*_0_	2.91	0.26	11.03	+
*b*_1_	0.03	0.16	0.17	−
*b*_2_	0.27	0.16	1.69	−
*b*_3_	0.81	0.16	4.98	+
*b*_11_	−0.31	0.24	1.31	−
*b*_22_	−0.22	0.24	0.94	−
*b*_33_	−0.51	0.24	2.13	−
*b*_12_	−0.01	0.23	0.05	−
*b*_13_	−0.54	0.23	2.37	−
*b*_23_	−0.12	0.23	0.54	−

+ significant; − insignificant.

**Table 9:** The coefficients of polynomials found for the viscosity of the PHUs.

*b*_i_		*s_b_*	*t_calc_*	Significance
*b*_0_	45.52	3.57	12.75	+
*b*_1_	−78.67	2.19	35.99	+
*b*_2_	43.32	2.19	19.82	+
*b*_3_	457.80	2.19	209.46	+
*b*_11_	−114.82	3.22	35.69	+
*b*_22_	114.20	3.22	35.50	+
*b*_33_	433.29	3.22	134.68	+
*b*_12_	−3.55	3.09	1.15	−
*b*_13_	−172.98	3.09	55.96	+
*b*_23_	96.17	3.09	31.11	+

+ significant; − insignificant.

**Table 10:** A comparison of actual and modeled properties of an optimized PHU.

	Viscosity [mPa·s]	Shear Strength [MPa]	*T_g_* [°C]
modeled	146	3.07	41.7
experimental	64	2.62	44.9

## Data Availability

The data presented in this study are available on request from the corresponding author.
